# Tópicos Emergentes em Insuficiência Cardíaca: Nova Era do Tratamento Farmacológico

**DOI:** 10.36660/abc.20201106

**Published:** 2020-11-01

**Authors:** Fabiana G. Marcondes-Braga, Felix J. A. Ramires, Estêvão Lanna Figueiredo, José Albuquerque Figueiredo, Luís Beck-da-Silva, Salvador Rassi

**Affiliations:** 1 Universidade de São Paulo Faculdade de Medicina Instituto do Coração do Hospital das Clínicas São PauloSP Brasil Instituto do Coração do Hospital das Clínicas da Faculdade de Medicina da Universidade de São Paulo (InCor.HCFMUSP), São Paulo, SP - Brasil; 2 Hospital do Coração São PauloSP Brasil Hospital do Coração (HCOR), São Paulo, SP - Brasil; 3 Instituto Orizonti Hospital Vera Cruz Belo HorizonteMG Brasil Instituto Orizonti e Hospital Vera Cruz, Belo Horizonte, MG - Brasil; 4 Universidade Federal do Maranhão São LuísMA Brasil Universidade Federal do Maranhão (UFMA), São Luís, MA - Brasil; 5 Hospital de Clínicas de Porto Alegre Porto AlegreRS Brasil Hospital de Clínicas de Porto Alegre, Porto Alegre, RS - Brasil; 6 Universidade Federal do Rio Grande do Sul Porto AlegreRS Brasil Universidade Federal do Rio Grande do Sul, Porto Alegre, RS - Brasil; 7 Universidade Federal de Goiás Hospital das Clínicas GoiâniaGO Brasil Hospital das Clínicas da Universidade Federal de Goiás, Goiânia, GO – Brasil

**Keywords:** Insuficiência Cardíaca, Tratamento Farmacológico, Insuficiência Cardíaca de Fração de Ejeção Reduzida

## Introdução

Nas últimas décadas, avanços no tratamento farmacológico e no uso de dispositivos implantáveis trouxeram mudanças no prognóstico de pacientes com insuficiência cardíaca de fração de ejeção reduzida (ICFEr). No entanto, ainda existe risco residual e alta morbimortalidade. Novas medicações, com ação em diferentes mecanismos fisiopatológicos, complementam a ação sobre o sistema neuro-humoral e remodelamento e seus benefícios ocorrem em adição à terapia padrão otimizada. A tabela 1 descreve os principais estudos sobre o tratamento medicamentoso da IC.

**Tabela 1:** Resumo dos principais estudos envolvendo tratamento farmacológico da insuficiência cardíaca

Estudos		População	Desfecho primário	NNT‡
**β-bloqueadores**				
CIBIS II^9^	Bisoprolol* 10 mg/d	2.647 pacientes NYHA III-IV FEVE ≤ 35% Seguimento: 16 m	Mortalidade por todas as causas RRR = 34%	18
MERIT HF^8^	Succinato de metoprolol* 200 mg/d	3.991 pacientes NYHA II-IV FEVE ≤ 40% Seguimento: 12 m	Mortalidade por todas as causas RRR = 34%	27
COPERNICUS^10^	Carvedilol* 25 mg 2xd	2.289 pacientes NYHA IV FEVE < 25% Seguimento: 11 m	Mortalidade por todas as causas RRR = 35%	15
**IECAs/BRAS**				
SOLVD^2^	Enalapril* 10 mg 2xd	2.569 pacientes NYHA CF II-IV FEVE ≤ 35% Seguimento: 37 m	Mortalidade de todas as causas RRR = 16%	22
CHARM^3^	Candesartana* 32 mg/d	2.028 pacientes NYHA CF II-IV FEVE < 40% Seguimento: 37 m	Mortalidade cardiovascular ou HIC RRR = 27%	14
**AMR**				
RALES^4^	Espironolactona* 25-50 mg/d	1.663 pacientes NYHA CF III-IV FEVE ≤ 35% Seguimento: 24 m	Mortalidade de todas as causas RRR = 30%	10
EMPHASIS^5^	Eplerenone* 25-50 mg/d	2.737 pacientes NYHA CF II FEVE ≤ 35% Seguimento: 21 m	Mortalidade cardiovascular ou HIC RRR = 37%	13
**INRA**				
PARADIGM-HF^6^	Sacubitril-Valsartana† 200 mg 2xd	8.442 pacientes NYHA CF II-IV FEVE < 40% / FEVE ≤ 35% Seguimento: 27 m	Mortalidade cardiovascular ou HIC RRR = 20%	21
**Vasodilatadores diretos**				
A-HEFT^11^	Hidralazina 225 mg/d + Dinitrato de Isossorbida* 120 mg/d	1.050 pacientes negros NYHA III e IV FEVE ≤ 35%, ou FEVE < 45% se DDVE > 6.5 cm ou > 2,9 cm/m2 Seguimento: 18 m	Morte por qualquer causa, primeira HIC e mudança na qualidade de vida Mortalidade total RRR = 43%	25
**Inibidores If**				
SHIFT^12^	Ivabradina* 5 – 7,5 mg 2xd	6.558 pacientes NYHA II - IV FEVE < 35% Ritmo sinusal / FC > 70 Seguimento: 23 m	Morte cardiovascular ou HIC RRR = 18%	26
**Digitálicos**				
DIG13	Digoxina* 0,25 mg/d	6.800 pacientes NYHA II - III FEVE < 45% Seguimento: 37 m	Mortalidade geral Ausência de redução	NA
**Inibidores SGLT2**				
DAPA-HF^14^	Dapagliflozina* 10 mg/dia	4.744 pacientes NYHA II-IV FEVE < 40% Seguimento: 18 m	Mortalidade cardiovascular ou HIC RRR = 26%	21
EMPEROR-Reduced^15^	Empagliflozina* 10 mg/dia	3.730 pacientes NYHA II-IV FEVE < 40% Seguimento: 16 m	Mortalidade cardiovascular ou HIC RRR = 25%	19
**Estimuladores da guanilato ciclase**				
VICTORIA^16^	Vericiguat* 10 mg/d	5.050 pacientes NYHA II, III ou IV FEVE < 45% Seguimento: 11 m	Morte cardiovascular ou primeira HIC RRR = 10%	24

* Versus Placebo.

† Versus Enalapril.

‡ NNT: definido para desfecho primário / morte por todas as causas no tempo total de seguimento.

iECA: inibidores da enzima de conversão da angiotensina; BRA: bloqueadores dos receptores de angiotensina II; HIC: hospitalização por INRA: inibidores da neprilisina e bloqueadores dos receptores de angiotensina II; BB: betabloqueadores; AMR: antagonistas mineralocorticoides; NYHA: New York Heart Association; IC: insuficiência cardíaca; FEVE: fração de ejeção ventricular esquerda; iSGLT2: inibidores do cotransporte de sódio e glicose 2; FC: frequência cardíaca.

### Terapia Padrão Otimizada

#### Sistema Renina-angiotensina-aldosterona (IECAs/ BRAs/ AMRs)

Estudos de desfechos clínicos importantes, como mortalidade e hospitalização, evidenciaram a fundamental importância do sistema renina-angiotensina-aldosterona (SRAA), com a atenuação da ação da angiotensina (AngII), utilizando os Inibidores da enzima de conversão da angiotensinaI (IECAs) ou os bloqueadores do receptor da AngII (BRAs), sendo esses últimos indicados em intolerantes aos IECAs.

Além da atenuação da ação da AngII, os antagonistas mineralocorticoides (ARMs) também se mostraram fundamentais na modulação do SRAA, tanto em pacientes mais sintomáticos (CF III-IV, NYHA) quanto naqueles com menos sintomas (CFII).

#### Inibição da Neprilisina (associada ao bloqueio do receptor de AngII)

Mais recentemente, a atenuação da ação deletéria da AngII, associada ao efeito protetor dos peptídeos natriuréticos, utilizando uma nova classe de fármacos, os inibidores da neprilisina e dos receptores da AngII (INRAs), cuja molécula atualmente disponível é o sacubitril/valsartana, mostrou-se superior aos IECAs, tanto na redução de mortalidade quanto de hospitalização por IC (HIC). Inicialmente, era indicada em substituição aos IECAs/BRAs apenas em pacientes ambulatoriais que se mantivessem sintomáticos (CFII-III, NYHA). Novos dados sustentam a possibilidade de início de tratamento com sacubitril/valsartana, ao invés de IECAs/BRAs, em pacientes com IC nova, assim como em pacientes ainda internados por IC descompensada após estabilização.

#### Bloqueio do Sistema Nervoso Simpático

Apesar dos avanços terapêuticos recentes, os β-bloqueadores (carvedilol, succinato de metoprolol e bisoprolol) continuam fundamentais no tratamento da ICFEr, por comprovadamente reduzirem sintomas, morte (por todas as causas, por IC e súbita) e hospitalizações em pacientes sintomáticos ou com disfunção ventricular assintomática.^8^^–^^10^ Devem ser iniciados em todos os pacientes, associados aos bloqueadores do SRAA, em doses reduzidas e tituladas àquelas utilizadas nos ensaios clínicos.

### Terapias farmacológicas adicionais

#### Nitrato/Hidralazina

A associação de nitrato e de hidralazina demonstrou ser efetiva na redução de desfechos sólidos, mortalidade total e HIC em pacientes autodeclarados negros. A associação pode ainda ser indicada para pacientes que apresentam piora da função renal e/ou hipercalemia em uso de IECA/BRA/INRA.

#### Ivabradina

A ivabradina inibe seletivamente a corrente I*f* no tecido do nó sinusal, reduzindo a frequência cardíaca (FC), que é um marcador de eventos em IC e um alvo terapêutico. Associa-se à redução do desfecho combinado de morte cardiovascular ou HIC em pacientes em ritmo sinusal, sintomáticos, com FC ≥ 70bpm e fração de ejeção ventricular esquerda (FEVE) ≤ 35%), sendo esse benefício basicamente por redução de HIC.

#### Digoxina

O uso de digoxina em pacientes com ICFEr foi avaliado na década de 90 e não se associou à redução de mortalidade em relação ao placebo. Houve, no entanto, redução das HIC..O efeito da digoxina e o seu lugar no tratamento contemporâneo da IC é desconhecido. Parece ser mais segura e efetiva na redução dos sintomas quando usada em baixas doses, guiada pelo nível plasmático e taxa de filtração glomerular(TFG).

### Inovações no Tratamento Farmacológico

#### Inibidores de SGLT2

Os benefícios dos iSGLT2 na redução de eventos cardiovasculares adversos maiores e de HIC em pacientes com diabetes tipo 2 (DM2) foram observados inicialmente com a empagliflozina. Subsequentemente, diferentes iSGLT2 também demonstraram redução de HIC em pacientes diabéticos. Diante desses achados, os iSGLT2 foram avaliados em pacientes com IC.

No DAPA-HF (Dapagliflozin and Prevention of Adverse Outcomes in Heart Failure), 4.744 pacientes com ICFEr foram randomizados para receber dapagliflozina ou placebo além da terapia padrão, sendo 41,8% com DM2. O desfecho primário de morte cardiovascular ou agravamento da IC foi significativamente menor no grupo dapagliflozina (26% de redução). Quando analisados separadamente, houve redução significativa tanto na morte cardiovascular (18% de redução) quanto na piora da IC (30% de redução), independentemente de DM2. Tais resultados revelam uma nova terapia para IC, já aprovada para essa finalidade.

O Empagliflozin Outcome Trial in Patients with Chronic Heart Failure and a Reduced Ejection Fraction (EMPEROR-Reduced) avaliou a empagliflozina *versus* placebo, além da terapia padrão, em 3.730 pacientes com ICFEr, sendo 50,2% com DM2. Os pacientes apresentavam IC mais grave do que aqueles no DAPA-HF, com média de FEVE de 27% contra 31%, sendo que mais de 70% dos pacientes tinham FEVE < 30%, além de maior nível mediano de NT-proBNP (1.907 vs 1.437 pg/mL). Houve redução de 25% do desfecho primário de morte cardiovascular ou HIC a favor da empagliflozina. Quando analisados separadamente, não houve redução de morte cardiovascular, resultado diferente daquele observado no DAPA-HF. Novamente, o benefício foi visto independentemente da presença de DM2.

Esses dados confirmam os resultados do DAPA-HF e reforçam a justificativa para o uso de iSGLT2 em pacientes com ICFEr, para redução dos sintomas, melhora da qualidade de vida, redução do risco de hospitalização e morte cardiovascular.

#### Estimuladores da Guanilato Ciclase Solúvel (GCs)

Vericiguat, um novo estimulador da GCs, aumenta a produção de guanosina monofosfato (GMP) por estimulação direta da GCs, através de um sítio de ligação independente de óxido nítrico (ON) e sensibiliza a GCs ao ON endógeno. Age suprindo o déficit relativo de produção de GMPc que ocorre na IC.

O ensaio clínico VICTORIA (Vericiguat in patients with reduced ejection fraction*)* alocou 5.050 pacientes com ICFEr, FEVE < 45%, CF II-IV NYHA, para receber vericiguat, via oral, ou placebo, em adição à terapia padrão. O desfecho primário, morte cardiovascular ou primeira HIC ocorreu em 35,5% *versus* 38,5% dos pacientes em favor do vericiguat (NNT = 24 em 11 meses). O benefício do desfecho composto deveu-se prioritariamente à redução de hospitalizações, não havendo benefício estatisticamente significativo em morte cardiovascular ou em mortalidade total.

Essa medicação tem potencial de integrar o grupo de medicações com efeito sobre sintomas e re-hospitalizações em pacientes com ICFEr, especialmente em pacientes com: hospitalizações frequentes, a despeito de terapia otimizada; pior função renal, já que o estudo considerava inclusão de pacientes com TFG > 15%; ou com intolerância a outras drogas, sendo contraindicada em concomitância com nitratos.

**Figura 1:**
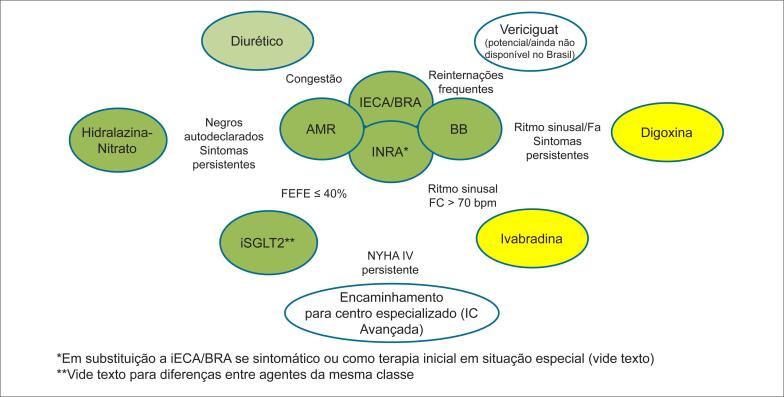
Abordagem farmacológica da Insuficiência cardíaca de fração de ejeção reduzida. iECA: inibidores da enzima de conversão da angiotensina; BRA: bloqueadores dos receptores de angiotensina II; INRA: inibidores da neprilisina e bloqueadores dos receptores de angiotensina II; BB: betabloqueadores; AMR: antagonistas mineralocorticoides; NYHA: New York Heart Association; IC: insuficiência cardíaca; FEVE: fração de ejeção ventricular esquerda; iSGLT2: inibidores do cotransporte de sódio e glicose 2; FC: frequência cardíaca; FA: fibrilação atrial.

Diante das novas evidências, dispomos de amplo arsenal terapêutico (Figura 1), capaz de impactar o prognóstico de pacientes com ICFEr. Uma vez iniciada terapia tripla, a otimização de doses e a individualização do tratamento, de acordo com o perfil do paciente, deve ocorrer precocemente, sabendo que há impacto em redução de mortalidade e HIC, quando acrescidas à terapia padrão.

### Lista de Participantes do Heart Failure Summit Brazil 2020 / Departamento de Insuficiência Cardíaca - DEIC/SBC

Aguinaldo Freitas Junior, Andréia Biolo, Antonio Carlos Pereira Barretto, Antônio Lagoeiro Jorge, Bruno Biselli, Carlos Eduardo Montenegro, Denilson Campos de Albuquerque, Dirceu Rodrigues de Almeida, Edimar Alcides Bocchi, Edval Gomes dos Santos Júnior, Estêvão Lanna Figueiredo, Evandro Tinoco Mesquita, Fabiana G. Marcondes-Braga, Fábio Fernandes, Fabio Serra Silveira, Felix José Alvarez Ramires, Fernando Atik, Fernando Bacal, Flávio de Souza Brito, Germano Emilio Conceição Souza, Gustavo Calado de Aguiar Ribeiro, Humberto Villacorta Jr., Jefferson Luis Vieira, João David de Souza Neto, João Manoel Rossi Neto, José Albuquerque de Figueiredo Neto, Lídia Ana Zytynski Moura, Livia Adams Goldraich, Luís Beck-da-Silva Neto, Luís Eduardo Paim Rohde, Luiz Claudio Danzmann, Manoel Fernandes Canesin, Marcelo Bittencourt, Marcelo Westerlund Montera, Marcely Gimenes Bonatto, Marcus Vinicius Simões, Maria da Consolação Vieira Moreira, Miguel Morita Fernandes da Silva, Monica Samuel Avila, Mucio Tavares de Oliveira Junior, Nadine Clausell, Odilson Marcos Silvestre, Otavio Rizzi Coelho Filho, Pedro Vellosa Schwartzmann, Reinaldo Bulgarelli Bestetti, Ricardo Mourilhe Rocha, Sabrina Bernadez Pereira, Salvador Rassi, Sandrigo Mangini, Silvia Marinho Martins, Silvia Moreira Ayub Ferreira, Victor Sarli Issa.
