# Supplementary material for: Tópicos Emergentes em Insuficiência Cardíaca: Nova Era do Tratamento Farmacológico
Source: Arq Bras Cardiol. 2020 Nov 1;115(5):956–60. [Article in Portuguese] doi: 10.36660/abc.20201106 (PMC8452188; doi:10.36660/abc.20201106)
Supplement: Translation

# Translation: Emerging Topics in Heart Failure: New Era of Pharmacological Treatment

**Authors:** Fabiana G. Marcondes-Braga, Felix J. A. Ramires, Estêvão Lanna Figueiredo, José Albuquerque Figueiredo, Luís Beck-da-Silva, Salvador Rassi

**Affiliations:** 1 São PauloSP Brazil Instituto do Coração do Hospital das Clínicas da Faculdade de Medicina da Universidade de São Paulo (InCor.HCFMUSP), São Paulo, SP - Brazil; 2 São PauloSP Brazil Hospital do Coração (HCOR), São Paulo, SP - Brazil; 3 Belo HorizonteMG Brazil Instituto Orizonti e Hospital Vera Cruz, Belo Horizonte, MG - Brazil; 4 São LuísMA Brazil Universidade Federal do Maranhão (UFMA), São Luís, MA - Brazil; 5 Porto AlegreRS Brazil Hospital de Clínicas de Porto Alegre, Porto Alegre, RS - Brazil; 6 Porto AlegreRS Brazil Universidade Federal do Rio Grande do Sul, Porto Alegre, RS - Brazil; 7 GoiâniaGO Brazil Hospital das Clínicas da Universidade Federal de Goiás, Goiânia, GO – Brazil

**Keywords:** Heart Failure, Pharmacological Treatment, Heart Failure with Reduced Ejection Fraction

## Introduction

In recent decades, advances in pharmacological treatment and implantable devices have impacted the prognosis of heart failure (HF) with reduced ejection fraction (HFrEF). However, there remains high residual risk to be addressed. New therapies with different pathophysiologic targets can enhance the action of medications on the neurohormonal system and remodeling, and these benefits occur in addition to those of standard medical therapy. Table 1 depicts the main results of randomized clinical trials on HF treatment.

**Table 1:** Summary of the main results of randomized clinical trials on heart failure treatment

Clinical trials		Population	Primary outcome	NNT‡
**β-blockers**				
CIBIS II^9^	Bisoprolol* 10 mg once daily	2647 patients NYHA III-IV LVEF ≤ 35% Follow-up: 16 mo	All-cause mortality RRR=34%	18
MERIT HF^8^	Metoprolol succinate* 200 mg once daily	3991 patients NYHA II-IV LVEF ≤ 40% Follow-up: 12 mo	All-cause mortality RRR=34%	27
COPERNICUS^10^	Carvedilol* 25 mg twice daily	2289 patients NYHA IV LVEF < 25% Follow-up: 11 mo	All-cause mortality RRR=35%	15
**ACEIs/ARBs**				
SOLVD^2^	Enalapril* 10 mg twice daily	2569 patients NYHA II-IV LVEF ≤ 35% Follow-up: 37 mo	All-cause mortality RRR=16%	22
CHARM^3^	Candesartan* 32 mg once daily	2028 patients NYHA II-IV LVEF < 40% Follow-up: 37 mo	Cardiovascular mortality or HHF RRR=27%	14
**MRAs**				
RALES^4^	Spironolactone* 25-50 mg once daily	1663 patients NYHA III-IV LVEF ≤ 35% Follow-up: 24 mo	All-cause mortality RRR=30%	10
EMPHASIS^5^	Eplerenone* 25-50 mg once daily	2737 patients NYHA CF II LVEF ≤ 35% Follow-up: 21 mo	Cardiovascular mortality or HHF RRR=37%	13
**ARNIs**				
PARADIGM-HF^6^	Sacubitril-valsartan† 200 mg twice daily	8442 patients NYHA II-IV LVEF < 40% / LVEF ≤ 35% Follow-up: 27 mo	Cardiovascular mortality or HHF RRR=20%	21
**Vasodilators**				
A-HEFT^11^	Hydralazine 225 mg once daily + Isosorbide dinitrate* 120 mg once daily	1050 black patients NYHA III-IV LVEF ≤ 35% or LVEF < 45% if LVDD > 6.5 cm Follow-up: 18 mo	All-cause mortality, first HHF, and quality of life All-cause mortality RRR=43%	25
**I*f* inhibitor**				
SHIFT^12^	Ivabradine* 5-7.5 mg twice daily	6558 patients NYHA II-IV LVEF ≤ 35% Sinus rhythm / HR >70 Follow-up: 23 mo	Cardiovascular mortality or HHF RRR=18%	26
**Digitalis**				
DIG^13^	Digoxin* 0.25 mg once daily	6800 patients NYHA II-III LVEF < 45% Follow-up: 37 mo	All-cause mortality No reduction	NA
**SGLT2 inhibitors**				
DAPA-HF^14^	Dapagliflozin* 10 mg once daily	4744 patients NYHA II-IV LVEF < 40% Follow-up: 18 mo	Cardiovascular mortality or HHF RRR=26%	21
EMPEROR-Reduced^15^	Empagliflozin* 10 mg once daily	3730 patients NYHA II-IV LVEF < 40% Follow-up: 16 mo	Cardiovascular mortality or HHF RRR=25%	19
**GC stimulators**				
VICTORIA^16^	Vericiguat* 10 mg once daily	5050 patients NYHA II-IV LVEF < 45% Follow-up: 11 mo	Cardiovascular mortality or first HHF RRR=10%	24

* Versus Placebo.

† Versus Enalapril.

‡ NNT: defined for the primary endpoint/all-cause mortality during follow-up.

ACEI: angiotensin-converting enzyme inhibitor; ARB: angiotensin II receptor blocker; HHF: hospitalization for heart failure; ARNI: angiotensin II receptor-neprilysin inhibitor; MRA: mineralocorticoid receptor antagonist; NNT: number needed to treat; NYHA: New York Heart Association; HF: heart failure; LVEF: left ventricular ejection fraction; SGLT2i: sodium-glucose cotransporter 2 inhibitor; HR: heart rate; GC: guanylate cyclase; RRR: relative risk reduction; LVDD: left ventricular end-diastolic diameter.

### Standard Medical Therapy

#### Renin-angiotensin-aldosterone System (ACEIs/ARBs/MRAs)

The fundamental importance of the renin-angiotensin-aldosterone system (RAAS) has been underscored in randomized controlled trials reporting attenuation of angiotensin II (AngII) action with the use of angiotensin-converting enzyme inhibitors (ACEIs) or AngII receptor blockers (ARBs), with the latter being indicated in patients who do not tolerate ACEIs. Mineralocorticoid receptor antagonists (MRAs) also play a key role in RAAS modulation in both more symptomatic (New York Heart Association [NYHA] class III-IV) and less symptomatic (NYHA class II) patients.

#### Neprilysin Inhibition Combined with AngII Receptor Blockade

More recently, a new drug class, the dual-acting AngII receptor-neprilysin inhibitor (ARNI), whose commercially available molecule is sacubitril/valsartan, combined the attenuation of AngII harmful action with the protective effect of natriuretic peptides and proved to be superior to ACEIs in reducing both mortality and hospitalization for HF (HHF). It was initially indicated to replace ACEIs/ARBs only in outpatients who remained symptomatic (NYHA class II-III). However, new data support the possibility of starting treatment with sacubitril/valsartan, instead of ACEIs/ARBs, in patients with new-onset HF as well as in hospitalized patients.

#### Sympathetic Nervous System Blockade

Despite recent therapeutic advances, beta-blockers (carvedilol, metoprolol CR/XL, and bisoprolol) remain essential in the treatment of HFrEF, as they are associated with a reduction in symptoms, death (all-cause mortality, sudden cardiac death, or death due to worsening HF), and hospitalization in symptomatic patients and in those with asymptomatic ventricular dysfunction.^8^^–^^10^ Beta-blockers, combined with RAAS inhibitors, should be initiated in all patients at reduced doses and then uptitrated to the doses used in clinical trials.

### Additional Medical Therapies

#### Hydralazine-isosorbide Dinitrate

Combined isosorbide dinitrate-hydralazine therapy showed a reduction in all-cause mortality and HHF in patients self-identified as black who had HF. This combination may also be used in patients with worsening renal failure or hyperkalemia with ACEI/ARB/ARNI use.

#### Ivabradine

A high resting heart rate (HR) is a risk factor for patients with HFrEF and a potential therapeutic target. Ivabradine is a selective sinus-node I*f-*channel inhibitor whose action results in HR lowering. In patients with HF, ivabradine reduced the combined endpoint of cardiovascular death or HHF in patients in sinus rhythm with HR > 70 bpm and left ventricular ejection fraction (LVEF) < 35%. The main benefit was reduced HHF.

#### Digoxin

In the 1990s, digoxin was evaluated in patients with HFrEF and showed no association with reduced mortality compared to placebo, but there was a significant reduction in HHF. The role of digoxin in contemporaneous HF treatment remains unknown. Its use at low doses appears to be safe and effective in improving symptoms if treatment is guided by plasma levels and glomerular filtration rate (GFR).

### Innovations in Pharmacological Treatment

#### Sodium-glucose Cotransporter 2 (SGLT2) Inhibitors

The benefits of SGLT2 inhibitors in reducing major adverse cardiovascular events and HHF in patients with type 2 diabetes (T2D) were initially observed with the use of empagliflozin. Subsequently, different SGLT2 inhibitors also showed a reduction in HHF in patients with diabetes. In view of these findings, SGLT2 inhibitors were evaluated in patients with HF.

In the Dapagliflozin and Prevention of Adverse Outcomes in Heart Failure (DAPA-HF) trial, 4744 patients with HFrEF were randomized to receive dapagliflozin or placebo in addition to standard therapy; of these, 41.8% had T2D. The primary outcome (a composite of cardiovascular death or worsening HF) was significantly lower in the dapagliflozin group (26% reduction). There was a significant reduction in both cardiovascular death (18% reduction) and worsening HF (30% reduction) when analyzed separately, regardless of the presence or absence of T2D. These results reveal a new therapy for HF, already approved for this purpose.

The Empagliflozin Outcome Trial in Patients with Chronic Heart Failure and a Reduced Ejection Fraction (EMPEROR-Reduced) evaluated empagliflozin vs placebo, in addition to standard therapy, in 3730 patients with HFrEF; 50.2% with T2D. Patients appeared to have more severe disease than those in the DAPA-HF trial, with a median LVEF of 27% against 31%. Also, more than 70% of patients had LVEF < 30% in the EMPEROR-Reduced trial and a higher median level of N-terminal prohormome of brain natriuretic peptide (NT-proBNP) (1907 vs 1437 pg/mL). There was a 25% reduction in the primary outcome (a composite of cardiovascular death or HHF) in favor of empagliflozin. Like in the DAPA-HF trial, the benefit was seen regardless of the presence or absence of T2D. However, different from the DAPA-HF trial, no reduction was observed in cardiovascular death when analyzed separately.

#### Soluble Guanylate Cyclase (sGC) Stimulators

Veriguat, a novel sGC stimulator, enhances the cyclic guanosine monophosphate (cGMP) pathway by directly stimulating sGC through a binding site, independent of nitric oxide (NO), and sensitizes sGC to endogenous NO. It acts by enhancing the relative insufficient production of cGMP, common in HF.

The Vericiguat Global Study in Subjects with Heart Failure with Reduced Ejection Fraction (VICTORIA) randomly assigned 5050 patients with HFrEF, LVEF < 45%, and NYHA class II-IV to receive vericiguat or matching placebo, in addition to standard therapy. The primary outcome (a composite of cardiovascular death or first HHF) was significantly less frequent in the vericiguat group (35.5%) than in the placebo group (38.5%), and the number needed to treat was 24 for 1 year. The main benefit within the composite endpoint was a reduction in HF hospitalization, with no statistically significant difference in cardiovascular or all-cause mortality.

This medication has the potential to be included in the group of HF medications with an effect on symptoms and rehospitalizations, especially in patients with frequent hospitalizations despite optimal medical therapy, patients with renal failure (the VICTORIA trial included patients with an estimated GFR > 15%), and those intolerant to other medications. Concomitant use with nitrates is contraindicated.

### Final Considerations

New therapeutic options have been developed, with a great impact on HF prognosis (Figure 1). In this new era of HF treatment, once standard medical therapy is initiated, new medications that reduce mortality and HHF may be started.

**Figure 1:** Pharmacological management of patients with heart failure with reduced ejection fraction (HFrEF). ACEI: angiotensin-converting enzyme inhibitor; ARB: angiotensin II receptor blocker; ARNI: angiotensin II receptor-neprilysin inhibitor; BB: beta-blocker; MRA: mineralocorticoid receptor antagonist; NYHA: New York Heart Association; HF: heart failure; LVEF: left ventricular ejection fraction; SGLT2i: sodium-glucose cotransporter 2 inhibitor; HR: heart rate.

### List of Participants of the Heart Failure Summit Brazil 2020 / Heart Failure Department - Brazilian Society of Cardiology

Aguinaldo Freitas Junior, Andréia Biolo, Antonio Carlos Pereira Barretto, Antônio Lagoeiro Jorge, Bruno Biselli, Carlos Eduardo Montenegro, Denilson Campos de Albuquerque, Dirceu Rodrigues de Almeida, Edimar Alcides Bocchi, Edval Gomes dos Santos Júnior, Estêvão Lanna Figueiredo, Evandro Tinoco Mesquita, Fabiana G. Marcondes-Braga, Fábio Fernandes, Fabio Serra Silveira, Felix José Alvarez Ramires, Fernando Atik, Fernando Bacal, Flávio de Souza Brito, Germano Emilio Conceição Souza, Gustavo Calado de Aguiar Ribeiro, Humberto Villacorta Jr., Jefferson Luis Vieira, João David de Souza Neto, João Manoel Rossi Neto, José Albuquerque de Figueiredo Neto, Lídia Ana Zytynski Moura, Livia Adams Goldraich, Luís Beck-da-Silva Neto, Luís Eduardo Paim Rohde, Luiz Claudio Danzmann, Manoel Fernandes Canesin, Marcelo Bittencourt, Marcelo Westerlund Montera, Marcely Gimenes Bonatto, Marcus Vinicius Simões, Maria da Consolação Vieira Moreira, Miguel Morita Fernandes da Silva, Monica Samuel Avila, Mucio Tavares de Oliveira Junior, Nadine Clausell, Odilson Marcos Silvestre, Otavio Rizzi Coelho Filho, Pedro Vellosa Schwartzmann, Reinaldo Bulgarelli Bestetti, Ricardo Mourilhe Rocha, Sabrina Bernadez Pereira, Salvador Rassi, Sandrigo Mangini, Silvia Marinho Martins, Silvia Moreira Ayub Ferreira, Victor Sarli Issa.
